# Health Promoting Behaviors, Health Needs and Associated Factors among Older Adults in Jordan: A Cross-Sectional Study

**DOI:** 10.30476/ijcbnm.2020.87493.1443

**Published:** 2021-04

**Authors:** Mohammad Rababa, Nahla Al Ali, Ayat Alshaman

**Affiliations:** 1 Department of Adult Health Nursing, Faculty of Nursing, Jordan University of Science and Technology, Irbid, Jordan; 2 Department of Community and Mental Health Nursing, Faculty of Nursing, Jordan University of Science and Technology, Irbid, Jordan

**Keywords:** Health behavior, Health promotion, Healthy Lifestyle, Older adults

## Abstract

**Background:**

Several factors affect older adults’ engagement in HPBs. This study aimed to examine HPBs, health needs, and associated factors among older adults in Jordan.

**Methods:**

A cross-sectional study was conducted on 220 older adults at one governmental and one university hospital, which were selected using convenience sampling for geographical closeness to the researchers. All older adults with no cognitive or communication problems who attended the outpatient clinics of the two hospitals from December 2018 to April 2019 were included in the study. This time period was chosen based on the convenience of the participants and researchers. Data were collected by An Arabic version of the Health-Promoting Lifestyle Profile (HPLP) and a demographic questionnaire. The Statistical Package for Social Science (SPSS) 25.0 software was used for the descriptive and inferential analysis of the study data. The level of significance was set at P<0.05.

**Results:**

The mean score of the total HPLP was 125.33±19.09. The marital status and educational level of the participants were associated with the total HPLP (P<0.001) in all its dimensions, except for the dimension of interpersonal relations. Participants with chronic diseases had lower scores than those without diseases for the total HPLP (P<0.001) in all the six dimensions. Family income was positively correlated with the dimensions of nutrition (P=0.007) and exercise (P=0.002).

**Conclusion:**

Despite the good overall mean score of older adults for total HPLP and some of its subscales, their levels of exercise and physical activity need to be promoted. The scores of older adults were related to various demographic and clinical factors.

## INTRODUCTION

The older adult population is a vulnerable group with unique healthcare challenges and needs. ^1^ Approximately 80% of older adults live with at least one psychological or physical comorbid problem worldwide. ^2^ Comorbid problems are considered the leading cause of death among older adults and account for two-thirds of all health costs. ^3^ In Jordan, about 86% of older adults have one psychological or physical comorbid problem, such as hypertension (HTN) (53%), Diabetes Mellitus (DM) (25%), high cholesterol level (30%), asthma (10%), heart diseases (13%), depression (22.5%), dementia (5%), and anxiety disorders (5.3%). ^4^

Lack of social support system is another highly prevalent problem among older adults, making them even more vulnerable than other age groups. ^5^ A systematic review of 128 studies conducted in the United States of America (USA), United Kingdom (UK), and Netherland showed that loneliness and social isolation were the most common social problems among older adults and were risk factors for many physical and mental health problems and increased mortality rates. ^5^ The high prevalence of malnutrition among older adults is also associated with diminished functional ability, muscle weakness, decreased immune system, poor wound healing, and increasing hospital readmission rates. ^6^ A recent study found that physical inactivity, poor diet, and smoking increase the risk of disability among older adults in France. It also found that people with all three unhealthy behaviors had more than a twofold increased risk of disability compared with those without such behaviors. ^7^

Therefore, many healthcare organizations started to highlight the concept of health promotion among older adults. World Health Organization (WHO) developed the strategies of health promotion and healthy aging in the first international conference, “Ottawa Charter,” in 1986. ^8^ HPBs were defined by Pender as “the desired behavioral endpoint or outcome of health decision making”. ^9^ Adopting HPBs, such as weight reduction, smoking cessation, physical activity, and stress management, is associated with improved quality of life, increased life expectancy, and decreased morbidity and mortality rates. ^9^ Pender’s health promotion model ^10^ was used to guide the current study to assess HPBs, health needs, and associated factors among older adults in Jordan. 

According to the Pender’s health promotion model, ^10^ factors contributing to HPBs are categorized as biological (e.g. age and gender), psychological (e.g. self-esteem and self-motivation), and socio-cultural (e.g. race/ethnicity, and socioeconomic status). Age was found to be negatively correlated with HPBs, as with advanced age, adherence to HPBs decreases. ^11^ A study conducted in the United Arab Emirates showed that the level of education and gender were significant predictors of HPBs among older adults. ^12^ Zanjani et al. found that males were more likely than females to be engaged in HPBs, such as stress management, exercise, social relation, and adequate nutrition. ^11 , 13^ Another study emphasized that social support significantly contributed to HPBs. ^13^

In Jordan, few studies were conducted on HPBs in particularly older adults. ^14^ Evidence showed that most Jordanian older adults still follow Western habits, such as poor eating habits, lack of activity, and life stress. ^15^ Studies on the difference in HPBs among Jordanian older adult groups according to their demographic characteristics, such as gender, marital status, income, level of education, showed conflicting findings. ^14^ Jordanian older adults encounter many public health challenges affecting their adherence to HPBs. According to the National Council for Family Affairs (NCFA(, approximately 78% of Jordanian older adults lost their jobs or were retired early due to physical and sensory disabilities. ^16^ The presence of chronic diseases among Jordanian older adults was one of the significant predictors of healthcare services utilization. ^17^ Therefore, assessment of HPBs and health needs among older adults is crucial for successful aging and better quality of life. This study aimed to examine the HPBs, health needs, and associated factors among older adults in Jordan. 

## MATERIALS AND METHODS

This cross-sectional study was conducted on a convenience sample of 220 older adults, who were referred to the outpatient clinics in a governmental or university hospital in Irbid, Jordan, during December 2018 to April 2019. These two hospitals are located in an ethnically and culturally homogenous area in Irbid city, Jordan. The hospitals were chosen due to convenience for geographic closeness to the researchers and because they have the target population of interest. The use of the outpatient clinics of local hospitals provides feasible access to community-dwelling older adults. Drawing from the same ethnic and cultural population limits the generalizability but controls some potential confounding effects. The time of data collection was chosen based on the convenience of the participants and researchers and to keep the study feasible and avoid some other confounding events that could occur if a longer time period was used. Participants over the age of 60 years with Jordanian ethnicity, and able to comprehend and sign the consent form were included in this study. Participants with cognitive impairments or communication deficits were excluded from the study. The sample size was determined based on Eshah ’s study, ^14^ and according to the following formula: considering the parameters; α=0.05, d=0.06, and σ=0.43. An additional 5% was added to overcome the refusal rate, yielding a total sample size of 220. 

 The Arabic version of the HPLP scale ^18^ based on the Pender’s health-promoting model was used in the present study. The scale consists of 47 items scored using 4 points Likert scale: 1=never, 2=sometimes, 3=often, 4=routinely. The total score of the HPLP ranges from 47 to 188, with higher scores indicating greater engagement in HPBs. The scale consisted of six dimensions of HPBs described as follows: self-actualization (11 items), interpersonal relations (7 items), nutrition (7 items), exercise (5 items), health responsibility (10 items), and stress management (7 items). The Content validity the scale was established by four experts in public health and nursing education. ^18^ The content validity index (CVI) of the Arabic HPLP was 0.90, indicating an acceptable level of content validity. ^18^ The construct validity of the Arabic HPLP was established by factor analysis and found similar to those of the English and Spanish versions. ^18^ The reliability of the HPLP was established previously among Jordanian people. ^14 , 15^ The Cronbach’s alpha coefficient of the sub-scales ranged from 0.60 to 0.89 and equaled 0.89 for the total scale, indicating the instrument’s high internal consistency. ^18^ In the current study, the internal consistency reliability (Cronbach’s alphas) for the total HPLP was 0.89; for the subscales it ranged from 0.67 (nutrition), 0.68 (stress management), 0.71(exercise), 0.73 (interpersonal relations), 0.74 (self-actualization), and 0.79 ( health responsibility).

Demographic characteristics, including gender, marital status, monthly income, level of education, chronic diseases, smoking status, and health insurance, were collected by asking the participants to fill out the demographic questionnaire. 

After obtaining ethics committee approval (IRB #832/2018) from the Institutional Review Board (IRB) committee of research on human at Jordan University of Science and Technology, permission was obtained from each hospital. The nurse in charge of outpatient clinics was contacted, and a list of potential participants with their contact information was obtained to facilitate the recruitment process. Written informed consent was obtained from the participants who agreed to participate in the present study during their clinic visits. All participants providing consents had an opportunity to ask their questions. The consent form emphasized the privacy protections on the part of the participants in this study. The voluntary nature of the study and confidentiality of the collected data were emphasized by the researchers. The collected data were not disclosed to anyone by the researchers without written consent from the participant. The researchers trained a research assistant holding a bechelor’s dgree in Nursing about how to collect the study data. The research assistant collected the relevant information from the older adults who agreed to participate through a structured face-to-face interview in the waiting room. The interview took an average of 25 minutes. Data were collected from December 2018 to April 2019.

 Data were analyzed using SPSS, Version 25.0 (Armonk, NY: IBM Corp). The level of significance was set at 0.05. The descriptive analysis (Mean, frequencies, standard deviation, and percentages) was used to describe the participants’ demographic characteristics. Pearson correlation was used to assess the correlation between the level of HPBs and participants’ monthly income. Independent t-test was also used to determine the difference the levels of HPBs between the study groups based on the participants’ sociodemographic characteristics, including gender, marital status, presence of health insurance, diagnosis of chronic disease, education level, and smoking. One-way ANOVA was used to examine the difference in the mean scores of HPLP and its subscales between the participants with different education levels. 

## RESULTS

The total number of participants who sufficiently completed the study questionnaires was 220 with a response rate of 95.6%. Of these participants, 146 (66.4%) were males, 74 (33.6%) were females, 178 (80.9%) were married, and 184 ‎(83.6%) had chronic diseases. The participants’ age ranged from 60 to 87 (66±6.186), their family income ranged from 40-3000 Jordanian Dinar (JD), and their educational level ranged from less than basic to the higher education level, with the majority (33.2%) achieving the secondary level. The results of Kolmogorov–Smirnov (P=0.33) and Shapiro–Wilk (P=0.54) tests were not statistically significant, indicating that data were normally distributed. The demographic characteristics of the participants are presented in Table 1.

**Table 1:** Sociodemographic characteristics of the participants (N=220)

Variables	N (%) a
Gender	
Male	146 (66.40)
Female	74 (33.60)
Marital status	
Married	178 (80.90)
Unmarried	42 (19.10)
Health insurance	
Yes	197 (89.50)
No	23 (10.50)
Diagnosis with chronic disease	
Yes	184 (83.60)
No	36 (16.40)
Smoking status	
Yes	85 (38.60)
No	135 (61.40)
Educational level	
Illiterate	35 (15.90)
Basic	45 (20.50)
Secondary	73 (33.20)
Diploma	24 (10.50)
Higher education	43 (19.50)

a Number and percentages

Table 2 displays the observed ranges, mean, and SD of HPBs. The total mean scores of HPLP was 125.33±19.09; the highest scores were in the dimension of the self-actualization (30.35±5.69) and the lowest was in the dimension of exercise (11.52±3.73). 

**Table 2:** Mean±SD score of total health promoting lifestyle profile and its subscales

HPLP^a^ scale	Mean±SD^b^	Range
HPLP total score	125.33±19.09	60-179
Self-actualization	30.35±5.69	14-44
Health responsibility	25.92±6.04	11-40
Nutrition	18.66±3.59	10-28
Exercise	11.52±3.73	5-20
Stress management	18.25±4.17	7-28
Interpersonal relation	20.61±4.15	7-28

a Health-promoting lifestyle profile

b Standard deviation

As seen in Table 3, the results of the independent sample t-test revealed no statistically significant differences between the female and male older adults in their total HPLP (P=0.30) and all HPBs dimensions (P>0.05). Also, no statistically significant differences were found between older adults with health insurance and those without it in the total HPLP (P=0.90) and all HPBs dimensions (P≥0.05). Marital status was significantly associated with the total HPLP (P<0.001), and all HPBs dimensions, except for the dimension of interpersonal relations (P=0.44). The results also revealed significant differences between older adults with chronic diseases and healthy ones in the total mean score of HPLP (P<0.001) and all six dimensions (P<0.05). Moreover, there was a significant difference between smoker and non-smoker older adults only in the self-actualization subscale (P<0.01). Smokers had a lower mean score of self-actualization than nonsmokers. 

**Table 3:** Relationship between the participants’ demographic characteristics and total score of Health promoting lifestyle profile and its subscales

		HPLP^a^	Self-actualization	Health responsibility	Nutrition	Exercise	Stress management	Interpersonal relation
		Mean±SD^b^	Mean±SD	Mean±SD	Mean±SD	Mean±SD	Mean±SD	Mean±SD
Gender	Male	126.88±18.24	30.44±5.61	26.08±5.71	18.66±3.73	11.73±3.60	18.60±4.27	20.81±4.05
	Female	23.45±20.66	30.19±5.88	25.61±6.66	18.74±3.34	11.09±3.96	17.58±3.90	20.23±4.35
	P value *	0.30	0.76	0.54	0.81	0.23	0.08	0.33
Marital status	Married	127.88±18.86	30.83±5.66	26.60±6.07	18.94±3.61	12.08±3.49	18.71±4.11	20.72±4.11
	Unmarried	114.52±16.22	28.33±5.43	23.07±5.01	17.50±3.33	9.12±3.79	16.33±3.86	20.17±4.36
	P value *	<0.001	0.01	<0.01	0.01	<0.001	<0.01	0.44
Chronic Disease	Yes	123.05±18.23	29.88±5.72	25.38±5.87	18.41±3.61	11.18±3.64	17.87±4.04	20.34±4.22
	No	136.97±19.38	32.81±4.92	28.72±6.18	19.97±3.27	13.25±3.72	20.22±4.29	22.00±3.52
	P value * ‎	<0.001	<0.01	<0.01	0.01	<0.01	<0.01	0.02
Smoking status	Yes	122.86±20.80	28.98±6.22	25.59±6.48	18.19±4.09	11.64±3.78	18.18±4.70	20.29±4.28
	No	126.88±17.83	31.22±5.17	26.13±5.76	18.96±3.23	11.44±3.70	18.30±3.80	20.81±4.07
	P value * ‎	0.12	<0.01	0.51	0.12	0.71	0.82	0.36
Health Insurance	Yes	125.27±19.10	30.61±5.59	25.89±6.03	18.58±3.47	11.39±3.77	18.11±4.13	20.69±4.09
	No	125.78±19.39	28.13±6.14	26.17±6.25	19.35±4.57	12.65±3.21	19.52±4.31	19.96±4.71
	P value* ‎	0.90	0.05	0.83	0.33	0.12	0.12	0.42

* Independent sample t-test;

a Health-Promoting Lifestyle Profile

b Standard deviation

Data in Table 4 presents the associations between the participants’ level of education and the dimensions of HPLP. One-way ANOVA with a Tukey Post Hoc test revealed significant differences between the participants’ groups based on the level of education in the total HPLP and all dimensions, except for the interpersonal relations (P=0.10). 

**Table 4:** Relationship between the mean score of health promoting lifestyle profile subscale and the education level of the participant

	Illiterate	Basic	Secondary	Diploma	Higher Education	F	P*
	Mean±SD^b^	Mean±SD	Mean±SD	Mean±SD	Mean±SD		
HPLP^a^	116.00±17.36	122.98±14.30	123.00±21.74	131.4±16.14	135.9±16.29	7.18	<0.001
Self-actualization	29.03±6.36	30.53±4.19	28.84±6.66	31.83±4.86	33.00±3.83	4.82	<0.001
Health responsibility	23.37±5.33	25.24±5.61	25.75±5.61	27.38±5.34	28.19±5.92	3.75	<0.001
Nutrition	17.86±3.86	17.58±3.03	18.33±3.80	20.29±2.45	20.12±3.46	4.93	<0.001
Exercise	9.57±3.76	10.44±3.36	11.81±3.62	12.54±3.97	13.16±3.18	6.57	<0.001
Stress management	16.60±4.77	18.27±3.16	18.03±4.70	19.08±3.67	19.51±3.45	2.73	0.03
Interpersonal relation	19.57±4.64	20.91±3.63	20.25±4.42	20.29±4.12	21.95±3.57	1.93	0.10

* One-way analysis of variance ANOVA;

a Health-Promoting Lifestyle Profile

b Standard deviation

Pearson correlation coefficient showed a significant positive correlation between the participant’s family income and the dimensions of nutrition (r=0.183, P<0.001) and exercise (r=0.203, P<0.001). As their income increased, older adults were more likely to have adequate and healthy nutrition and do physical exercise.

## DISCUSSION

The results of this study showed that older adults in Jordan had various levels of practicing HPBs, with the highest level being in self-actualization, health responsibility and interpersonal relations, and the lowest level in exercise, nutrition, and stress management. 

The finding of the highest mean score in the self-actualization dimension was supported by a recent Turkish study. ^19^ According to Maslow’s hierarchy of the needs theory, basic human needs are met during adulthood, and older adults strive more toward self-actualization in their later life. ^20^ Maslow proposed that mentally healthy individuals follow a path named a growth motivation, which allows them to move up through the hierarchy of needs and finally reach the state of self-actualization. Moreover, the low mean score of physical activity indicates that older adults did not regularly engage in physical activity. ^21 - 24^ This was supported by previous studies, which found that about two-thirds of Jordanian are socialized to be less engaged in physical activity. ^22^ Age-related musculoskeletal changes such as decreased muscle and bone mass and reduced muscle strength lead to a decline in physical activity. ^25^ High comorbid burden among older adults is considered another reason for their low level of exercise. ^14^ In our study, 84% of the participants reported having one or more chronic diseases, which, in turn, limited their physical activity levels. The low level of physical activity reported in the current study could be attributed to the Jordanian culture, which consider practicing outdoor walking with training outfits, especially for female older adults acceptable. ^23^

This study also revealed that older adults had an acceptable level of health responsibility. Health responsibility is improved with advanced age, as described by Pender ^10^ in the HPLP model. According to the model, older adults’ sense of health responsibility is developed in later life when they have enough time after retirement to take care of themselves more than anytime else. Moreover, Mirghafourv et al. found that health responsibility is positively associated with the amount of social support provided by spouses and family members. ^26^ In our study, about 81% of the participants were married and were living with their spouses, which explains their acceptable level of health responsibility. Older adults in a Jordanian Islamic culture are very respectful and considered wise persons who are called in for any family and political negotiations. 

Furthermore, the finding that older adults had an acceptable level of interpersonal relations is consistent with the results of Dahlheim-Englund et al. ^27^ Contrary to our result, Beliran and Legaspi found that older adults have poor interpersonal relations. ^28^ This inconsistency could be attributed to the tight social bonds and stable family structure in Jordan. ^29^ Social support has a vital role in enhancing the social interaction of older adults and helping them to adhere more to HPBs, including interpersonal relations behaviors. ^27^

The finding that the participants reported poor nutritional behaviors is consistent with the previous studies. ^30^ Mosleh and Darawad found that about two-thirds of the Jordanian population had poor adherence to the low-fat diet. ^31^ The prevalence of poor nutritional behaviors among older adults could be attributed to age-related changes, such as sensory impairment, low appetite, dental problems, decreased gastric acid secretion, and associated vitamin deficiencies. Also, older adults are vulnerable to malnutrition due to their low monthly income, limiting the variety of their food options. ^32^ In Jordan, individuals suffer from many economic barriers, including low income and high unemployment rates that impede their ability to afford healthy food. ^32^ In our sample, the average family monthly income was only 423 JD (553 USD). 

The older adults in the current study had a reduced level of stress management. This finding was supported by previous studies ^11 , 33^ that emphasized that older adults had difficulty managing their stressful situations. The high prevalence of physical and psychosocial stressors among older adults paralleled with diminished independence and compromised coping mechanisms contributes to poor stress management. ^11 , 33^

Various factors were associated with the level of different dimensions of HPBs, such as the participants’ marital status, educational level, family income, smoking status, and the presence of chronic disease. The study finding that the married participants have higher mean scores on the HPLP and all HPBs than unmarried counterparts is in the same line with those of a previous study. ^34^ The findings of the current study supported the positive impact of being married on older adults’ health behaviors. Married participants receive different types of support, including emotional, social, and instrumental supports that lead to healthy health behaviors. ^35^

The findings of the current study showed that older adults having chronic diseases were more likely to have a lower mean score on the total HPLP. These findings are consistent with those of a previous Jordanian study that found older adults with no coexisting chronic diseases had better self-actualization, stress management skills, and interpersonal relationships and adequate nutrition. ^15^ Jordan has a unique prevalence of some chronic diseases associated with low HPLP in comparison to the neighboring countries. ^15^ Chronic diseases like DM, HTN, and coronary artery diseases were considered significant risk factors for unhealthy lifestyle and practices and physical disabilities. ^15^ The findings of a significant association between older adults’ smoking status and their scores on the self-actualization subscale is consistent with that of a recent study ^14^ which reported that smoking was associated with unhealthy habits, and smokers had less health responsibility and were less likely to adopt HPBs. 

The finding that the level of education had a statistically significant relationship with all HPLP subscales except interpersonal relations, with highly educated older adults being more likely to better adhere to HBPs was supported by previous studies. ^33 , 34 , 36 - 38^ These findings could be attributed to the fact that educated people know the importance and benefits of engagement in HPBs, and they have a better access to different health promotion resources. ^34^ Also, educated people are more aware of the negative consequences of unhealthy lifestyle and practices on their health. ^37^

The current study found significant differences between the participants based on their family income in terms of nutrition and exercise behaviors; older adults with higher income had higher levels of nutrition and exercise. Previous studies reported a similar finding. ^15 , 37 , 39^ For example, Shahrokhi et al. found that higher-income families had a higher nutrition subscale score, indicating a better nutritional status. Families with higher income had a wide variety of food choices.40 The positive relationship between exercise behaviors and income could be attributed to the fact that higher-income individuals have more time designated for leisure activities and outdoor exercises. ^41^ Moreover, older adults with low socioeconomic status receive less information about HPBs; therefore, they are less engaged in HPBs. ^42^

Health-promoting lifestyles of older adults are now a top priority in nursing practice to reduce premature death and enhance the quality of life for older adults. This study is the first among older adults in Jordan that assessed the HPBs and its associated factors. The finding of this study is the first step in identifying the health-promoting needs of older adults. These results of the study can help the nurses in developing effective health promotion programs to promote their quality of life. The current study would be a powerful message to the health policymakers that the health promotion programs in Jordan needs to be tailored to meet unmet health-promoting needs of older adults. 

This study had several limitations. The cross-sectional design does not permit the inference of causality. Participants in this study were recruited using convenience sampling method, which limits the generalizability of the findings to other groups. Thus, random sampling methods should be used in future research to have a more heterogeneous group and identify the differences among the subgroups. The Health Promotion Model described by Pender and used in this study suggested that individual characteristics and experiences, prior related behaviors (perceived benefits of action, perceived barriers to action, perceived self-efficacy, and activity-related affect), and personal factors interacted with each other and had direct ‎and indirect effects on the likelihood of engagement in health-promoting behaviors. The current study does not address all these factors; further interventional studies are still needed in this area.

## CONCLUSION

Despite the good overall mean score of older adults in total HPLP and the dimensions of self-actualization, health responsibility, and interpersonal relations, the level of exercise and physical activity among them was poor. The scores of older adults were related to various factors, including marital status, level of education, presence of chronic disease, smoking status, and family income. Future interventional studies are needed to target the health-promoting needs of older adults and improve their quality of life. Replication studies with larger sample size and in other clinical settings or other neighboring countries are recommended. 
